# Effectiveness of alternative approaches to integrating SDOH into medical education: a scoping review

**DOI:** 10.1186/s12909-022-03899-2

**Published:** 2023-01-11

**Authors:** Nehal Nour, David Stuckler, Oluwatobi Ajayi, Mohamed Elhassan Abdalla

**Affiliations:** 1 School of Medicine, University of Limerick, Faculty of Education & Health Services, Garraun, Castletroy, V94 T9PX Co. Limerick, Ireland; 2 Dondena Center for Research On Social Dynamics and Department of Social & Political Sciences, Bocconi University, 4 Via Roentgen 20136, Milan, Italy

**Keywords:** Social determinants of health, Curricula, Medical students, Medical schools, Teaching methods, Curriculum content

## Abstract

**Background:**

There is increasing recognition of including social determinants of health (SDOH) in teaching for future doctors. However, the educational methods and the extent of integration into the curriculum vary considerably—this scoping review is aimed at how SDOH has been introduced into medical schools' curricula.

**Methods:**

A systematic search was performed of six electronic databases, including PubMed, Education Source, Scopus, OVID (Medline), APA Psych Info, and ERIC. Articles were excluded if they did not cover the SDOH curriculum for medical students; were based on service-learning rather than didactic content; were pilot courses, or were not in English, leaving eight articles in the final study.

**Results:**

The initial search yielded 654 articles after removing duplicates. In the first screening step, 588 articles were excluded after applying inclusion and exclusion criteria and quality assessment; we examined 66 articles, a total of eight included in the study.

There was considerable heterogeneity in the content, structure and duration of SDOH curricula. Of the eight included studies, six were in the United States(U.S.), one in the United Kingdom (U.K.) and one in Israel. Four main conceptual frameworks were invoked: the U.S. Healthy People 2020, two World Health Organisation frameworks (The Life Course and the Michael Marmot's Social Determinants of Health), and the National Academic of Science, Engineering, and Medicine's (Framework For educating Health Professionals to Address the Social Determinants of Health).

In general, programs that lasted longer appeared to perform better than shorter-duration programmes. Students favoured interactive, experiential-learning teaching methods over the traditional classroom-based teaching methods.

**Conclusion:**

The incorporation of well-structured SDOH curricula capturing both local specification and a global framework, combined with a combination of traditional and interactive teaching methods over extended periods, may be helpful in steps for creating lifelong learners and socially accountable medical school education.

## Introduction

There is a growing interest in teaching social determinants of health (SDOH) curricula in medical schools to provide future physicians with the appropriate skills to assess, recognise and manage non-health barriers to health care access. The World Health Organization (WHO) defines SDOH as the avoidable non-medical factors influencing health outcomes, including where people are born, age, live, work and play. Poverty, for example, is linked to poorer access to health care services, unaffordability of medications, unhealthy nutritional choices, and unhealthy environmental living conditions – all of which negatively impact health status [1]. The WHO website states that the SDOH account for over 55% of variations in health outcomes [2].

Although the impact of SDOH on health outcomes is tremendous, physicians currently receive little training about how they can impact their patients and clinical practices. One recent survey conducted in 12 European Union (E.U.) medical institutes, representing 20,000 enrolled medical students, found that only one-third of the surveyed medical institutes provided SDOH curriculum to improve physicians' cultural competencies and their interaction and understanding of patients' diverse needs, cultural backgrounds. Few medical schools had any evaluation or monitoring of SDOH curricula, making it difficult to ascertain which were effective [3, 4]. Hence there is a growing interest in teaching social determinants of health (SDOH) in medical schools to provide future physicians with the appropriate skills to assess, recognise and manage non-health barriers to health care access.

There can be said to be a lack of research covering the actual integration of SDOH into medical school curricula and which of the alternative types of didactic methods could be used for more holistic teaching approaches. Various frameworks to deliver SDOH training exist; for example, the WHO Conceptual Framework for teaching SDOH is based on three components; education, community and organisation. This calls for doctors to engage during the learning process to formulate abstract concepts and reflect on the acquired knowledge (so-called 'experiential learning') in a supportive organisational environment to complement traditional desk-based education [5–7].

To address this gap, a systematic review is performed investigating how SDOH is taught at medical schools worldwide. This review creates a guide to the various SDOH teaching methods at medical schools and the curriculum content applied by these institutes. We map the main characteristics of the existing SDOH curricula: the conceptual frameworks used, the extent to which programmes integrate experiential learning and alternative didactic methods, and the evaluation/outcomes of curricula in improving physician competencies on SDOH.

## Methods

### Search strategy

A scoping review strategy was adopted to provide a comprehensive and transparent review. A systematic scoping search of published literature covering social determinants of health coursework integrated into medical school curricula worldwide was performed. All steps of the study conducted adhered to the PRISMA-ScR guidelines [8]. Following Peters and colleagues framework [9], the population was medical school students, including graduates and undergraduates; the concept was the curriculum content presented for teaching SDOH, and the context was the medical schools worldwide.

We searched six databases on May 20, 2021; (PubMed, Scopus, OVID (Medline), APA Psych Info, ERIC and Education Source), covering December 2010 to May 2021. The keywords selected were; social determinants of health, teaching, and medical school. Table 1 describes the permutations of each search term to ensure broad coverage. Where applicable, such as in PubMed and Ovid Medical Subject Heading terms and subject heading for "social determinants of health", which captured multiple definitions of SDOH, were employed. Also, we searched two grey literature databases (DART-Europe-E-thesis Portal and LENUS/the Irish Health Repository). Finally, we undertook citation searches to identify other papers for inclusion.

**Table 1:** Keyword search for the SDOH curricula

**Ovid MEDLINE search strategy (Literature search Covered till May 2021)** 1. Social determinants of health.mp or exp "Social Determinants of Health"/ 2. (Social determinants of health* or sdoh).mp 3. 1 or 2 4. exp Curriculum/ or exp Clinical Competence/ or exp Educational Measurement/ or exp Students, Medical/ or exp Education, Medical, Undergraduate/ or exp Education, Medical/ or medical education*.mp. or exp Education, Medical, Graduate/ 5. (curriculum* or medical education* or medical students* or medical schools*).mp 6. 4 or 5 7. 3 and 6 8. limit 7 to last 11 years
**PubMed search Strategy ( Literature search Covered till May 2021)** (("Social Determinants of Health"[Title/Abstract] OR "SDOH"[Title/Abstract]) AND ("curriculum"[Text Word] OR "teaching"[Text Word]) AND ("medical school"[Text Word] OR "medical schools"[Text Word])) AND (2010:2021[pdat])
**Scopus search strategy (Literature search Covered till May 2021)** ( TITLE-ABS-KEY ("Social determinants of health") OR TITLE-ABS-KEY (sdoh) AND KEY (curriculum OR curricula OR teaching OR learning) AND KEY ("medical student" OR "medical student" OR "medical education" OR "medical school" OR "medical schools")
**Education Source search strategy (Literature search Covered till May 2021)** AB ("social determinants of health" or "determinants of health" or sdoh) OR TI ("social determinants of health" or "determinants of health" or sdoh AND TX "medical education" or "medical school" or "medical students" or "medical curriculum" or "medical student education" AND (TX ("medical education" or "medical school" or "medical students" or "medical curriculum" or "medical student education")) AND (TX ( curriculum or curricula or instruction or teaching or learning)) Limiters—Published Date: 20,100,101–20,211,231
**APA PsychInfo search strategy (Literature search Covered till May 2021)** TI ( social determinants of health or determinants of health or sdoh) OR AB ( social determinants of health or determinants of health or sdoh) AND TX medical education or medical school or medical students or medical curriculum or medical student education or clinical education AND ((TX ( medical education or medical school or medical students or medical curriculum or medical student education or clinical education))
**ERIC international Search strategy (Literature search Covered till May 2021)** Ab("Social determinants of health") OR ab(sdoh) OR ti("Social determinants of health") AND (curriculum* or education*) AND medical*Published Date: 2010–2021

After removing duplicates, these papers were exported to Rayyan to undergo a blinded screening and eligibility stage independently by (N.N. and O.A.)

Two reviewers (N.N. and O.A.) performed the eligibility step, and in case of disagreements, a third reviewer resolved disputes about inclusion/exclusion criteria to reach a final inclusion decision.

### Inclusion/exclusion criteria

Articles were deemed eligible for inclusion if they evaluated SDOH curricula for undergraduate or graduate medical students. This included inter-professional SDOH programs that included medical students. Studies had to contain formal SDOH curriculum content to qualify for inclusion and describe teaching methods and approach employed. Articles were excluded if they focused on trainees, clinicians, nursing, dental, and pharmacy teaching rather than medical students. Studies were also excluded if: they did not contain sufficient information regarding the curriculum content and the learning; they did not focus exclusively on SDOH teaching.

### Data synthesis and analysis

The main characteristics of each curriculum were detailed, including the program title, length, layout, enrolment, educational methods, teaching concepts, the level of program implementation, and the learning competencies. Data from the eight included studies were extracted to an Microsoft Excel sheet, and key information about the authors, country of origin, year of publication, published journal and year of publication was included. We also extracted evaluation and success criteria for each program.

### Quality assessment tool

The Medical Education Research Study Quality Instrument (MERSQI) was selected for quality appraisal of the included articles. The appraisal tools assessed the articles over six domains, study design, sampling, type of data, the validity of the evaluation, data synthesis and outcome. Two reviewers (N.N and O.A) performed the assessment separately. The median score for the included articles was 11, seven out of the eight articles scored above 10 overall [10].

## Results

**Fig. 1:**
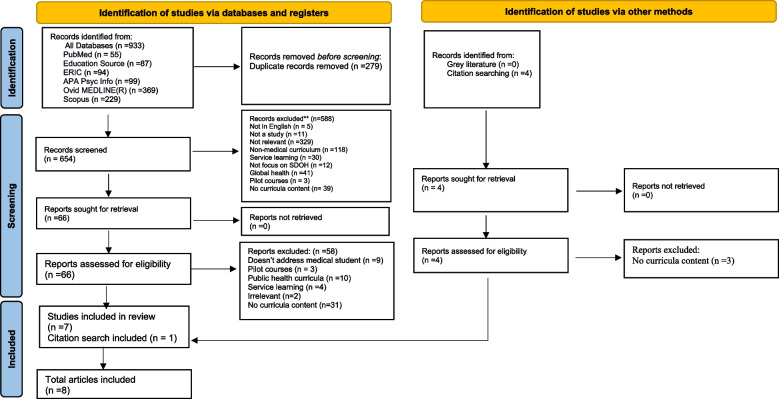
PRISMA flow diagram for the systematic scoping study on SDOH medical school curricula. From: Page MJ, McKenzie JE, Bossuyt PM, Boutron I, Hoffmann TC, Mulrow CD, et al. The PRISMA 2020 statement: an updated guideline for reporting systematic reviews. BMJ 2021;372:n71. https://doi.org/10.1136/bmj.n71

An initial search was performed through the six databases. The full keyword search yielded an initial 933 articles imported into the Endnote X9 reference manager. These articles were from the following sources: PubMed (*n* = 55), Education Source (*n* = 87), ERIC (*n* = 94), APA PsycInfo (*n* = 99), Ovid MEDLINE (*n* = 369), and Scopus (*n* = 229). After removing duplicates based on EndNote's find duplicate function and a hand search for duplicates (*n* = 279), 654 articles remained. Figure 1 depicts the PRISMA flow diagram for study inclusion.

In the first screening step, were a total of 588 articles excluded. Exclusion criteria were; not relevant (*n* = 329), did not cover medical curricula (*n* = 118), covered SDOH as applied to global health but not in the country of study (*n* = 41), were based on service-learning and not didactic content (*n* = 30), did not focus on SDOH (*n* = 12), not a study (*n* = 11), not in English (*n* = 5). Lastly, pilot courses were excluded (*n* = 3), and articles that did not provide sufficient information to evaluate the SDOH curricula (*n* = 39) were removed, leaving 66 articles for eligibility.

In this step, 66 full articles were examined and included employing the WHO definition of SDOH. A total of 58 articles were excluded because they were concerned with work-based learning in the community and not a structured curriculum (*n* = 4), insufficient curriculum details (*n* = 31), addressed non-medical students (*n* = 9), and studies related to public health curricula focusing on the prevention of infectious and chronic diseases rather than tackling the barriers of healthcare services (*n* = 10). Additionally, studies deemed irrelevant (*n* = 2) were identified and excluded. Studies that evaluated pilot courses (*n* = 3) were excluded as this study aimed to examine the formal curricula integrated into medical schools.

None of the records searched through the grey literature search were eligible for inclusion. The last search from the six included databases and the citation search of the reference lists yielded eight articles for inclusion in the scoping review. An additional manual search through the reference lists of these included articles yielded one further article which met eligibility criteria. The last search from the six included databases and the citation search of the reference lists yielded eight articles for inclusion in the scoping review.

### Overview of SDOH curricula

Table 2 provides an overview of each SDOH curriculum, and its primary feature. Of the eight curricula included in the review, six were from medical schools in the United States [11–16], one from the United Kingdom (U.K.) [17], and one from Israel [18]. Seven programs were aimed at medical students [11, 12, 14–18], and only one curriculum was an inter-professional program covering medical students and other health professionals, including medical, nursing, pharmacy school, public health students, and social work students [13].

**Table 2:** Summary table of the eight articles curricula content, structure, and the learning competencies of SDOH curricula

Study	Medical School	Program title	Program enrolment	Program structure	Program content	Program Length	Educational method(s)	Learning Competencies	Quality assessment reviewer 1	Quality assessment reviewer 2
Denizard-Thompson et al. 2021 [16]	United States (The Wake Forest School of medicine)	The health equity curriculum	Mandatory	Two days of simulation training 15 min session three times weekly for three weeks for the student's reflection, learning tasks, and group discussions	Module(1): Internal medicine and poverty/access to care Module(2): Psychiatry and food insecurity Module(3): Paediatrics and educational disparities Module(4): Obstetrics and gynaecology and women, infant health Module(5): Anaesthesiology and Implicit bias in pain Module(6): Family medicine and Transportation Module(7): Surgery and Environment/discharge planning Module(8): Neurology and Social network Module(9): Emergency medicine and housing The first 4 modules only contained community-based learning activities	Full-year for third year medical student	1. Didactic online or in-person 2.Experiential Learning 3. Reflective assignments and presentations	• Inter-professional learning experience • Critical thinking • Community engagement and exposure to diversity in realistic situations • Recognition of the community priorities and the impact of health outcomes • Reflective skills	15	14.5
Rockey et al. 2021 [11]	United States (Mayo Clinic Alix School of Medicine)	Student-run clinic	Mandatory	The clinic runs weekly over two and half days	Students take an initial assessment of the patients, then present to the physician, where he prescribes any further investigation or prescriptions needed	Full year for Second-year medical students	Experiential Learning	• Community engagement and exposure to diversity in realistic situations • Inter-professional experience and working with a multidisciplinary team • Recognition of the community priorities and the impact of health outcomes • Understanding the responsibilities of healthcare physicians towards patient's care • Basic health screening skills	12.5	13
Sagi et al. 2020 [18]	Israel (Azrieli Faculty of Medicine at Bar-Ilan University)	Etgar course *	Mandatory	A full-day introductory session Four tutorials within the clinical rotations Home visits within one week of discharge and follow up the phone within two weeks of the home visit Reports for their home-visits experience	Lectures and simulation-based training Tutorials include case simulation for patients to help recognise the SDOH Home-visit post-discharge, using a semi-structured report to evaluate the barriers for healthcare in underprivileged areas Planning a discharge plan and liaison with any services required	Full year for third and the fourth-year students	1. Didactic 2.Experiential Learning	• Realistic care experience • Early recognition of the healthcare equity barriers through home visits • Experience of community service with the broader context of SDOH • Reflective skills	12.5	12
Moffett et al. 2019 [12]	United States (New Jersey Medical School)	Social Determinants of Health course	Mandatory	Two orientation sessions for a small group of students Three learning activity stages over 4 weeks	15–20 min orientation session twice at the start to set the program layout and at the end for the student's reflection and oral presentations Learning activity(1): students-patients interview regarding patient's condition, their reflection on the hospital process starting from the E.D, social aspects and the discharge plan Learning activity(2): Small group discussions to generate research plan for each patient interviewed, explore SDH factors and offering solutions presented with PowerPoint presentation Learning activity(3): Oral presentation as a team facilitated by the faculty member to present the suitable plan and reflection	Four weeks for fourth-year medical students	1. Didactic 2. Experiential learning 3. Reflective	• Inter-professional workplace learning experience • Reflection skills • Recognition of the community priorities and the impact of health outcomes • Ability to apply this knowledge for appropriate referrals to relevant resources • Critical thinking	9.5	9
Gostelow et al. 2018 [17]	U.K (University College London Medical School)	Social determinants of the health curriculum	Mandatory	Online Self-paced learning for one week 90 min simulated scenarios discussions with a facilitators	The online self-directed learning consists of reading, videos like TED talks and small quizzes The discussion sessions with the simulated patients enable students to explore more into the social history, and pauses are made to highlight the main points regarding the health advocacy and health equity barriers	Full-year for fourth-year medical students	"Flipped classroom learning": pre-class reading or videos, followed by in-class case-based discussion, tutorials or simulation Collaborative learning	• The ability to understand health equity barriers in the U.K • Recognise the suitable interventions to overcome those barriers at various levels • Understanding the concepts of lifestyle drift and LifeCourse Concept • Recognition of the role of healthcare in reducing health inquiries on a local and global level • Recognise the suitable interventions to overcome those barriers at various levels • Apply the acquired knowledge to decrease health inquiries and apply health advocacy	10	9.5
Addy et al.2015 [13]	United States (University of South Carolina)	Interprofessional Education Program	Elective	Three live meetings Six web-based modules completed individually or with small group The six modules, integrated into the comprehensive courses at medicine and nursing schools, presented as independent pharmacy, public health, and social work schools	Module(1): introduction to inter-professional learning, team collaboration and patient safety Module(2): The roles of each disciplines in the health system towards patients Module(3): Innovation approach suggested by the student to improve healthcare Module(4): Cultural variation and its impact on healthcare decision, and cultural believes and communications Module(5): a devoted movie and reading to related topics Module(6): Case analysis and plan management to overcome healthcare barriers and students' reflection on the entire course	Variable according to each discipline .Medicine (first year) 2.Nursing 3. Pharmacy 4. Public Health 5. Social Works 6. Other disciplines	1. Didactic 2. Experiential learning	• The values of Inter-professional workplace experience • Roles of each health discipline toward the patients • Cultural competency • Identifying, analysis and planning for barriers regarding health equity	11	11
Gonzalez et al. 2015 [15]	United States (Albert Einstein College of Medicine in Bronx, New York)	Health Disparities elective	Elective	13 sessions, each one lasts for one and half hour Eight sessions are focusing on health disparities, and five sessions focusing on advocacy skills	Three sessions: Introductory of the health disparities Three sessions: Focusing on the factors contributing to the health disparities One practical clinical session: cultural competency skills practising such as open-ended questions, management methods, bias recognition, and management Five sessions: Advocacy skills, community perspectives on health disparities	Three months for first-year medical student	1. Didactic 2.Reflective assignments and presentations 3.Experiential Learning	• Legislative visits experience and community engagement • Collaborative learning • Recognition of the community priorities and the impact of health inequity on health outcomes • Creating advocacy skills and patient-doctor relationship skills and Writing and interview skills with simulated cases • Overcome the future health disparities factors	10.5	11.5
Drake et al. 2017 [14]	United States (Tulane University School of Medicine)	Social Contexts in Medicine	Elective	Six seminars/one and half hours each Four home visits(minimum) Three mentorship sessions Reflection exercise	Six seminars include: An introduction of the SDOH, Healthcare barriers and the infrastructure, Implicit bias of the healthcare providers, Interprofessional health responsibilities, and SDOH context Home-visit- kit and interview skills. a minimum of four home visits, each visit lasts about one to one and half hours, where the second-year student accompanies the first-year students to explore the healthcare barrier, connect with the patients on a social level, identify the suitable interventions and apply the basic health screening practices Mentorship sessions with one physician mentor and four students for reflection and discussion on the experience and the possible solutions for the health equity barriers	Eighteen months for first and second-year medical students	1. Didactic 2.Experiential Learning 3. Support and guidance learning	• Inter-professional workplace experience • Roles of each health discipline toward patients • Cultural competency • Identifying, analysis and planning for barriers regarding health equity within the local community • Basic health screening skills	12.5	12

Etgar* is a Hebrew word that means "challenge" is an abbreviation for literacy, support, and a bridge between medicine and society

A three-step review process was undertaken covering the structure of each curriculum (such as whether it was mandatory or not, the duration of the program), its content (the conceptual framework employed, which didactic methods were included, and the primary learning competencies focused on) and lastly whether the program was evaluated.

### Structure and content of SDOH curricula

Five medical schools included the SDOH curricula as a mandatory module [11, 12, 16–18], whereas three had it as an elective course [13–15]. The included programs varied in duration and timing during medical school training. Five were integrated over an entire academic year [11, 14, 16–18]; one of the five programs lasted 18 months (with a six-month preparation phase), and the remaining three varied between three and four months [12, 13, 15]. Regarding timing, four SDOH curricula were for third and fourth-year medical students at the beginning of the clinical clerkship [12, 16–18]. The final three programs focused on the first- and second-year medical students [11, 14, 15]. The remaining inter-professional program was integrated at different levels according to each school module design, so the timing of the course was variable [13].

All programs were structured based on a cited public health framework. The U.S. medical curricula [13, 14] were based on the United States public health department's *Healthy People 2020* objectives, the overarching 10-year strategic plan for eliminating health disparities [19, 20]. The main objectives of the U.S. initiative are eliminating health disparities related to socioeconomic conditions, gender, age, race, disability, sexual preference, or environmental status. These objectives can be achieved by improving the health status on a national level, promoting health equities for all age groups, increasing the awareness of the public sector regarding SDOH, working on intersectoral levels to enhance practices, and providing measurable indicators for health level improvement. *Healthy people 2020* captures 12 SDOH related topics, including health access, education, preventive Medicine, environmental condition, violence, sexual health, nutrition and physical health, maternal health, mental health, oral health, drug abuse, and smoking.

Two programmes drew upon two different WHO frameworks [17, 18]; the U.K. medical school *SDOH curriculum* [17] adopted the WHO *Life Course model* [21] which identifies the physical and social risk factors during various stages of life from prenatal to middle age, impacting health outcomes in later life*.* This model educates health professionals regarding the relationship between socioeconomic conditions and health inequalities. The *Etgar course* [18] from Israel adopted Michael Marmot's The Social Determinants of Health guidance [22], explaining ten solid points that link the social structure to the patient's health outcome. This guidance was an initiation of the WHO urban health centre to work as guidance for the public and policymakers.

The *Health equity curriculum* [16] at the *Wake Forest School of Medicine* is based on the National Academic of Science, Engineering, and Medicine's Framework For educating Health Professionals to Address the Social Determinants of health which recommends incorporating SDOH teaching over three domains; education, community, and organisations collaboration. The education domain comprises four areas, collaborative learning, experiential learning, integrated curriculum, and continuing professional [5]

The *Interprofessional course* at the* University of South Carolina* [13] integrated multiple frameworks. Specifically, it incorporated the Society of General Internal Medicine's Disparities Task Force guidelines for health disparities education, which covers the racial health disparities and the required knowledge to understand, assess, and recognise the barriers to health inequities. The American Academy of Paediatrics; and The Midwest Academy Manual for Activists frameworks were used to guide the organisational social work implemented in the curricula [23, 24].

*The student-run clinic program* at the *Mayo Clinic Alix School of Medicine* [11] and *the emergency clerkship* course from the New Jersey Medical School [12] stated that both curricula' accreditation using the Liaison Committee on Medical Education guidance. However, the framework designing for the SDOH curricula was not listed [25].

The method of delivering the SDOH courses also varied. Most of the curricula were delivered via group tutorials, sessions or group discussions within a classroom or clinical rotations. Three courses [13, 14, 18] used a combination of two teaching modalities: experiential learning and didactic. Another three courses [12, 16] used the same approach adding the student's reflection as a writing assay or oral presentation third modality. On the other hand, *the student-run clinic course* used the experiential learning method through the weekly student-run clinic [11]. Lastly, *the U.K. SDOH curricula* applied the innovative flipped classroom method, which includes pre-class learning resources and classroom discussion to enhance that knowledge [17].

The eight medical school curricula had diverse educational objectives. These varied considerably but tended to have a standard set of competencies: the ability to assess and recognise SDOH related health barriers according to each defined framework, interprofessional skills, representing the core competency of collaborative learning and communication. The programmes also sought to cultivate reflective skills, leadership and teamwork expertise. Teaching the students the ability to identify, analyse and evaluate the related issue or so-called” Critical thinking” was guided only by two programs [14, 22]. The eight medical programs learning competencies are detailed in Table 2.

### Evaluation and outcomes of the SDOH curricula

All the included curricula were evaluated for the knowledge, the gained competencies, and students' confidence to work with underserved populations. Yet, none of the studies assessed the impact of the student's knowledge on the patient's health outcomes. The evaluations were all performed with online surveys taken pre-and post-curriculum. Two of the eight programs also performed semi-structured interviews to evaluate the course [11, 17].

The eight programs improved the student's knowledge of SDOH concepts and implications on health outcomes; three programs [11, 15, 16] boosted the student's confidence level in dealing with social factors. One program [12] improved the ability to recognise the SDOH elements. Another program [13] conveyed interprofessional collaboration outcomes on students learning process.

Looking across programs, the highest-rated modalities according to students' self-assessment across the eight programs were the group discussions and the community engagement, which featured realistic patient-centred care experiences.

The analysis of each curriculum showed the following. *The Wake Forest School of Medicine curricula* [16] was evaluated based on three cohorts of 314 students. These cohorts included: the students who received the entire course (nine modules), the shorter course (three modules) and those who did not receive any teaching. The evaluation found significant improvements in the student's confidence and knowledge regarding SDOH through engagement within the emergency department. Knowledge was found to be retained for one year after the exposure to the longitudinal curricula. The results showed no difference between curricula of three to nine modules. The assessment represented the importance of incorporating the curriculum into the clinical clerkship years. The students will be confident to engage with patients and the thriving community partnership to identify the areas of need.

Similarly*, the Tulane University elective Curriculum* evaluation [14] was carried out three times, pre-and post-curricula and for the students who didn't receive the elective curricula. The evaluation, which involved 58 students, represented the increase of the students' awareness regarding SDOH through the community-based service and their wellness to work with the underserved population in the future. however, it showed the need for implementing early seminars for pre-clinical engagement to improve the acquired knowledge.

*The Student-run clinic curriculum at the Mayo Clinic Alix School of Medicine* [11] evaluation showed students' confidence to work with an underdeveloped population increased. The evaluation (*N* = 90 students) demonstrated the disparate outcomes related to the stigma reinforcement of the disadvantaged patients, the tension from dealing with patients in the early clinical years, and the various degrees of commitment to the self-directed learning aspect of the curricula.

The *Etgar course curriculum *[18] at *Azrieli Faculty of Medicine* at *Bar-Ilan University *evaluated the post-home visit surveys of 177 students. The analysis showed that home visits helped increase the student's awareness of the broader social context of the health inequities of their patients. The curricula enabled the students to explore the complexity of SDOH related factors in a realistic environment; however, the students reported that organising the visits and household language barriers were significant challenges.

The *SDOH curriculum* [12] at the *New Jersey Medical School *evaluated 56 students. After the course, online reflection showed increased recognition of the students' SDOH related factors and the ability to apply this knowledge in their practice. However, the evaluation reported that increasing the engagement with an experienced facilitator and more interactive learning activities will significantly impact the students' learning process.

The *SDOH curricula* [17] at *University College London* were evaluated using the 'flipped classroom method' through an online survey and semi-structured interviews. The evaluation involved 289 students and revealed an increase in students' perspectives regarding the social factors and their implication on their practice. Yet, the student's feedback favoured the discussion session over the taught part of teaching.

The evaluation of the *Inter-Professional curriculum* [13] at the* University of South Carolina* via pre and post-program survey showed enhancement of the students' knowledge regarding interprofessional collaboration between various disciplines. The evaluation, which involved 500 students, revealed that creating more interactive learning modalities will improve the learning impact.

A pre and post-program survey used to evaluate the *Health disparities elective curricula *[15]. The evaluation indicated that their knowledge and confidence regarding SDOH improved significantly, and it is now being proposed as a mandatory course.

## Discussion

Our review of eight medical school curricula found considerable variation in how SDOH was integrated into medical school curricula. Six of those had mandatory SDOH requirements. The programmes drew primarily on WHO SDOH frameworks [21, 22], the U.S. *Healthy People 2020* framework [20] and the National Academic of Science, Engineering, and Medicine's framework [5]. The best performing programmes for improving medical students' knowledge and awareness about SDOH appeared to be for longer durations than a few short months [11, 14, 16–18]. Students ratings indicated they most enjoyed community engagement and group discussions which allowed experiential learning rather than classroom-based didactic methods [12, 13, 15–18]. Several essential gaps were found in most of programs' evaluations; only one program focused on evaluating the ability of the students to retain the gained competencies after one year of finishing the program [16]. Students also voiced that SDOH training would be helpful prior to engage with real patients during their training [11–18].

### Study limitations

Our research has several limitations. First, our study excluded curricula that incorporated SDOH into other modules. This could potentially overlook effective and necessary modalities for integrating SDOH into the medical curriculum. Second, our search employed the WHO's definition of the SDOH term, as we did not include search terms like "health inequity" or "health equity" to draw specifically upon the WHO's well-established identification of the SDOH. However, when we included additional terms to capture SDOH, such as 'poverty' and 'living conditions, we did not capture different research papers on medical school SDOH curricula.

Third, there were limitations arising from the included studies themselves. Specifically, we only found studies in high-income countries like the U.S., the U.K., and Israel medical schools. It is possible that low- and middle-income countries have not published evaluations or descriptions of their SDOH curricula. Future research would be needed to identify these unpublished or grey literature evaluations of SDOH curricula. Ideally, we could have also evaluated differences between elective and mandatory courses, but unfortunately, in several cases, whether the course was obligatory could not be ascertained from publicly available information.

Despite these limitations, our study has several strengths. To our knowledge, this is the first systematic appraisal of how SDOH is integrated into medical school and the relative effectiveness of these programmes. Our findings also corroborate expert judgements about SDOH competencies. For example, in an influential study by Mangold et al. 2019 [26], the authors concluded that integrating SDOH teaching in medical schools as a longitudinal curriculum, not just during clinical rotations or pre-clinical period only, would better promote understanding of the intersectional relationship between health outcomes and social factors.

### Implications for future research and practice

Our research identifies several directions for future research. There is a clear need for better collaboration between the medical schools, the community partners and acknowledgment of the limitation and barriers [27]. Ideally, this would include a needs assessment of the local community and provide a mechanism for community partners to play a role in designing the SDOH curriculum.

Our research has several important implications for how best to integrate SDOH into medical school curricula. First, it revealed that multiple conceptual frameworks could be applied and adapted to local specificities, even though they capture the SDOH-related barriers differently [5, 20–22].

Secondly, programs that lasted longer and followed medical students longitudinally appeared to perform better than shorter duration programmes. This was especially important for equipping students with skills and competencies to apply SDOH in clinical settings. Nevertheless, shorter duration programs did significantly improve students' knowledge about SDOH [11, 14, 16–18].

Thirdly, most curricula relied on one or two methods to deliver the SDOH concepts. The teaching modalities varied between programs, with a predominance of didactic and experiential learning, which relies on students' engagement, reflection and application of this knowledge. The traditional lecture teaching method should take part in the preparatory stage for the course. Our finding demonstrates greater effectiveness when combining conventional and interactive teaching methods is employed. These interactive methods include the 'flipping teaching' technique, mentorship and realistic patient care experience on the students' knowledge and understanding [17]. Each program should be integrated with combined teaching modalities such as collaborative learning, experiential learning, integrated curriculum, and continuing professional to reinforce the SDOH concepts and create lifelong learners [5].

Fourthly, although the literature regarding teaching SDOH is increasing, published articles involving interprofessional collaboration are scarce [28]. It is essential to address other health professionals, not only physicians, via interprofessional coursesAs Lathrop stated, collaboration with various allied health professionals who lead SDOH teaching, such as nurses, is essential for promoting and addressing health equity [29]. Reducing the barriers of health inequities requires the collaboration of the whole health professional sector for a holistic approach and sustainable impact.

Finally, overall the programme evaluations were weak. They tended to focus on student knowledge; the greater focus should be placed on creating lifelong learners and the actual impact on patients' health outcomes.

Although still in the early stages, these initial findings show the great potential and promise for including SDOH in the medical curriculum. The benefits of combining teaching methods and incorporating various domains that capture the local specification with a global framework to create lifelong learners are promising. This will be an essential strategy to prepare the next generation of doctors and medical leaders to address health disparities and create socially accountable physicians.

## Data Availability

Datasets used and analysed during the study are available from the corresponding author upon reasonable request*.*

## References

[1] Social determinants of health. https://www.who.int/health-topics/social-determinants-of-health#tab=tab_1. Accessed 13 Jan 2022.

[2] https://www.who.int/hac/donorinfo/callsformobilisation/ethiopia_resmob.pdf?ua=1. 2020.

[3] Sorensen J, Norredam M, Suurmond J, Carter-Pokras O, Garcia-Ramirez M, Krasnik A (2019). Need for ensuring cultural competence in medical programmes of European universities. BMC Med Educ.

[4] Lewis JH, Lage OG, Grant BK, Rajasekaran SK, Gemeda M, Like RC (2020). Addressing the Social Determinants of Health in Undergraduate Medical Education Curricula: A Survey Report. Adv Med Educ Pract.

[5] National Academies of Sciences, Engineering, and Medicine. A framework for educating health professionals to address the social determinants of health. p. 3–11. 27854400

[6] Solar O, Irwin A. A conceptual framework for action on the social determinants of health. WHO Document Production Services; 2010.

[7] Doobay-Persaud A, Adler MD, Bartell TR, Sheneman NE, Martinez MD, Mangold KA (2019). Teaching the Social Determinants of Health in Undergraduate Medical Education: a Scoping Review. J Gen Intern Med.

[8] C. Tricco A, Lillie E, Zarin W, K. O’Brien K, Colquhoun H, Levac D, et al. PRISMA Extension for Scoping Reviews (PRISMA-ScR): Checklist and Explanation. Ann Intern Med. 2018. 10.7326/M18-0850. 10.7326/M18-0850 30178033

[9] Peters MDJ, Godfrey CM, Khalil H, McInerney P, Parker D, Soares CB (2015). Guidance for conducting systematic scoping reviews. Int J Evid Based Healthc.

[10] Cook DA, Reed DA (2015). Appraising the quality of medical education research methods: the Medical Education Research Study Quality Instrument and the Newcastle-Ottawa Scale-Education. Acad Med J Assoc Am Med Coll.

[11] Rockey NG, Weiskittel TM, Linder KE, Ridgeway JL, Wieland ML (2021). A mixed methods study to evaluate the impact of a student-run clinic on undergraduate medical education. BMC Med Educ.

[12] Moffett SE, Shahidi H, Sule H, Lamba S. Social determinants of health curriculum integrated into a core emergency medicine clerkship. MedEdPORTAL. 2019;15:10789. 10.15766/mep_2374-8265.10789 PMC6354789 30800989

[13] Addy CL, Browne T, Blake EW, Bailey J (2015). Enhancing interprofessional education: Integrating public health and social work perspectives. Am J Public Health.

[14] Drake C, Keeport M, Chapman A, Chakraborti C (2017). Social contexts in medicine: a patient-centered curriculum empowering medical students to provide contextualized care. MedEdPORTAL.

[15] Gonzalez CM, Fox AD, Marantz PR (2015). The evolution of an elective in health disparities and advocacy: description of instructional strategies and program evaluation. Acad Med J Assoc Am Med Coll.

[16] Denizard-Thompson N, Palakshappa D, Vallevand A, Kundu D, Brooks A, DiGiacobbe G (2021). Association of a Health Equity Curriculum With Medical Students’ Knowledge of Social Determinants of Health and Confidence in Working With Underserved Populations. JAMA Netw Open.

[17] Gostelow N, Barber J, Gishen F, Berlin A (2018). Flipping social determinants on its head: Medical student perspectives on the flipped classroom and simulated patients to teach social determinants of health. Med Teach.

[18] Sagi D, Spitzer-Shohat S, Schuster M, Rier D, Rudolf MCJ (2020). Learning social determinants of health through a home visiting course in the clinical years. Patient Educ Couns.

[19] Koh HK, Blakey CR, Roper AY (2014). Healthy People 2020: a report card on the health of the nation. JAMA.

[20] Koh HK, Piotrowski JJ, Kumanyika S, Fielding JE (2011). Healthy people: a 2020 vision for the social determinants approach. Health Educ Behav.

[21] Our work: life course. www.who.int. Available from: https://www.who.int/our-work/life-course.

[22] Wilkinson RG, Marmot MG (2003). Social determinants of health: the solid facts.

[23] Ross PT, Wiley Cené C, Bussey-Jones J, Brown AF, Blackman D, Fernández A, Fernández L, Glick SB, Horowitz CR, Jacobs EA, Peek ME (2010). A strategy for improving health disparities education in medicine. J Gen Intern Med..

[24] Organizing for Justice · Midwest Academy. https://www.midwestacademy.com/training/organizing-social-change/. Accessed 30 Jan 2022.

[25] LCME Accreditation. AAMC. https://www.aamc.org/services/first-for-financial-aid-officers/lcme-accreditation. Accessed 30 Jan 2022.

[26] Mangold KA, Bartell TR, Doobay-Persaud AA, Adler MD, Sheehan KM (2019). Expert Consensus on Inclusion of the Social Determinants of Health in Undergraduate Medical Education Curricula. Acad Med.

[27] Hubinette M, Dobson S, Scott I, Sherbino J (2017). Health advocacy. Med Teach.

[28] Molitor  WL, Naber  A, Cleveland  T, Regnerus  C, Wesner C, Zimney  K (2021). Exploring Interprofessional Activities that Address Poverty, Social Determinants of Health, Homelessness, and Chronic Pain. Health Interprofessional Pract Educ.

[29] Lathrop B (2013). Nursing Leadership in Addressing the Social Determinants of Health. Policy Polit Nurs Pract.

